# Points-based physical activity: a novel approach to facilitate changes in body composition in inactive women with overweight and obesity

**DOI:** 10.1186/s12889-018-5125-2

**Published:** 2018-02-17

**Authors:** Adrian Holliday, Alice Burgin, Elyzabeth Vargas Fernandez, Sally A. M. Fenton, Frank Thielecke, Andrew K. Blannin

**Affiliations:** 1 School of Sport, Exercise and Rehabilitation Sciences, University of Birmingham, Edgbaston, Birmingham UK; 2 Institute for Sport, Physical Activity & Leisure, Leeds Beckett University, Leeds, UK; 3 Institute of Sport and Exercise Science, University of Worcester, Worcester, UK; 4 T2 Bene Ltd. Thielecke Consulting, Bettenstrasse 60a, 4123 Allschwil, Switzerland

**Keywords:** Inactive, Sedentary behavior, Exercise, Body weight, Food intake, Spill-over

## Abstract

**Background:**

Physical activity (PA) interventions for the promotion of weight-management may benefit from increased choice and flexibility to overcome commonly-perceived barriers to PA. The aim of this study was to investigate the effects of a novel “points-based” approach to PA on body composition in inactive women, who are overweight or obese.

**Methods:**

Seventy-six overweight or obese, inactive women were randomly allocated to one of three conditions: ‘Points-based’ PA (PBPA; 30 “PA points”•week^− 1^), Structured exercise (StructEx; 150 min moderate-intensity exercise•week^− 1^) or control (CONT; continue habitual inactive lifestyle) for a 24-week intervention. PA points for activities were adapted from MET values, and 30 points was equivalent to 150 min of brisk walking. Measures of body composition (dual-energy x-ray absorptiometry) and anthropometry were obtained at weeks 0, 4, 12 and 24. Self-report activities were recorded weekly, with objective measures of PA (tri-axial accelerometry) and self-report measures of food intake obtained at weeks 0 and 24.

**Results:**

Fifty-eight women completed the study and provided data for primary outcomes. Of these, *n* = 41 and *n* = 19 provided data for food intake and objectively assessed PA. Mixed-design ANOVAs demonstrated that those in PBPA achieved a significant weight-loss at 24 weeks of − 3.3 ± 5.9 kg (− 3.4 ± 7.1%, *p* = 0.004). Waist circumference was reduced in PBPA at 24 weeks (− 2.8 ± 4.6 cm), compared with CONT (+ 2.1 ± 6.6 cm, *p* = 0.024). There was a trend for greater reductions in fat mass for those in PBPA vs. CONT (− 2.3 ± 4.6 kg vs. + 0.1 ± 2.0 kg, *p* = 0.075). Android fat was reduced in PBPA at both 12 weeks (− 6.1 ± 12.6%, *p* = 0.005) and 24 weeks (− 10.1 ± 18.4%, p = 0.005), while there was a trend for greater reductions in visceral adipose tissue in PBPA (− 5.8 ± 26.0%) vs. CONT at 24 weeks (+ 7.8 ± 18.3%, *p* = 0.053). Body composition, body weight and waist circumference were unchanged in StructEx. There were trends for increases in light-activity and reductions in sedentary time in PBPA. There was a trend for a reduction in daily energy intake of − 445 ± 564 kcal (*p* = 0.074), and a significant reduction in daily fat intake (*p* = 0.042) in PBPA.

**Conclusion:**

A “points-based” approach to physical activity appears to be an effective strategy for inducing modest reductions in body weight and body fat in inactive women with overweight and obesity.

**Trial registration:**

NCT02020239. Registered 12th December 2013.

**Electronic supplementary material:**

The online version of this article (10.1186/s12889-018-5125-2) contains supplementary material, which is available to authorized users.

## Background

Trends in adult body mass index (BMI) show a consistent increase in the global prevalence of obesity in men (3.2% to 10.8%) and women (6.4% to 14.9%) between 1975 and 2014 [1]. The World Health Organisation cites overweight and obesity as the 5th leading risk factor for mortality worldwide [2]. With such health implications allied with a considerable financial burden on health services [3], effective weight-management strategies are essential.

The importance of physical activity (PA) for weight-management is currently a topic of some contention. Most commonly, PA is accumulated through participation in structured exercise (i.e., purposeful, structured physical activity with the objective of maintaining or improving a component of fitness). Typically, weight-loss interventions consisting of exercise alone, without caloric reduction result in modest reductions in body weight [4, 5]. However, greatest weight-loss is achieved with a combination of exercise and diet [5, 6], and data underlines the efficacy of structured exercise and lifestyle PA for avoidance of weight-gain and for weight-loss maintenance [6–10]. Further, in comparison with dietary interventions, exercise interventions result in more favourable changes in body composition and reductions in fat mass relative to total body weight [11, 12]. Nevertheless, uptake of, and adherence to structured exercise programmes remains low [13–15]. For example, Edmunds et al. [13] found that only 51% of individuals who were overweight or obese successfully adhered to a 3-month exercise prescription programme. Similarly, Wiblur and colleagues [15] reported middle-aged, inactive women completed just 64% of the 96 walking exercise sessions in a 24-week programme. Hence, there remains need for effective as well as efficacious exercise and PA interventions [9].

Women represent a particularly inactive population. Recent data indicate that just 4% of women in England, U.K., engage in recommended levels of objectively assessed moderate-to-vigorous physical activity (MVPA) [16]. Still, despite the high prevalence of inactivity, 76% of women indicate they want to be more active [16]. The discrepancy in these statistics suggests there are barriers to PA engagement for this population. Accordingly, PA interventions are required which seek to address such barriers in order to facilitate the uptake and maintenance of PA among women [9].

Recent investigation would suggest two prominent perceived barriers to adoption and maintenance of PA among women: perceived lack of time [17] and lack of motivation [18, 19]. Consequently, it would seem desirable to develop time-efficient PA interventions that are compatible with every-day life [7], and do not require the structuring of exercise into perceived busy daily routines. It may also prove preferable for interventions to afford the exerciser choice and flexibility with regards to the manner in which PA is accumulated. Increased perceptions of autonomy, which can be achieved through offering greater choice and flexibility, is proven to increase intrinsic motivation and promote the uptake of, and adherence to PA behaviour [20, 21]. In addition, greater choice and flexibility, with the focus being on physical activity rather than structured exercise, may help overcome barriers of lack of enjoyment of, and low self-efficacy for exercise [22], the latter being particularly relevant for those who are overweight or obese [23, 24]. Previous research comparing structured exercise with lifestyle PA programmes has reported comparable favourable changes in physical activity and weight loss [25]. Still, research exploring intervention approaches that facilitate perceptions of autonomy and foster intrinsic motivation, may further enhance the effectiveness of PA programmes.

The aim of this study was therefore to investigate the effect of a novel points-based approach to PA on body weight and body composition in inactive middle-aged women, who were overweight. It was hypothesised that a points-based PA intervention (i.e., using PA points to provide choice and flexibility with regards to the accumulation of PA) would result in greater reductions in body weight, waist circumference and fat loss, compared with a traditional “structured” exercise intervention (30 min of moderate intensity exercise, 5 days per week). It was expected that the effectiveness of the intervention would be mediated by greater total PA engagement in the points-based PA condition.

## Methods

### Study design, Randomisation & Setting

The study utilised a between-subject, randomised control trial design, with participants allocated in a randomized manner to one of three groups for a 24-week study period: a points-based physical activity condition (PBPA); structured exercise condition (StructEx); a waiting-list control condition (CONT). Randomisation of participants (1:1:1 ratio) occurred at the individual level. An equal number of plain, sealed envelopes, each indicating either PBPA, StructEx or CONT, were placed in a box by a member of the research team, and participants drew an envelope to select their randomised condition. All study testing was conducted at the School of Sport, Exercise & Rehabilitation Sciences, University of Birmingham. Ethical approval was gained from the Solihull NRES Committee (Protocol number 13WM0331). This study was registered at clinicaltrials.gov (NCT02020239). The study was conducted and is reported in accordance with the CONSORT 2010 Statement.

### Sample size

To identify an approprirate sample size, an a priori sample size calculation was conducted using G* Power software [26]. Based on previous studies demonstrating fat mass or body weight reductions of > 5% [27–30] calculations revealed a necessary sample of between 33 and 54 participants to detect between-group differences of medium-large effect (f = 0.35–0.45, power = 0.8, α = 0.05). Due to the free-living nature of the present study and the number of primary outcome measures, and allowing for a 20% drop-out rate, a sample of 75 participants was sought. This sample size (total and for each level of the independent variable) is in line with a number of previous studies in the field [31–33].

### Participants

**Fig. 1:**
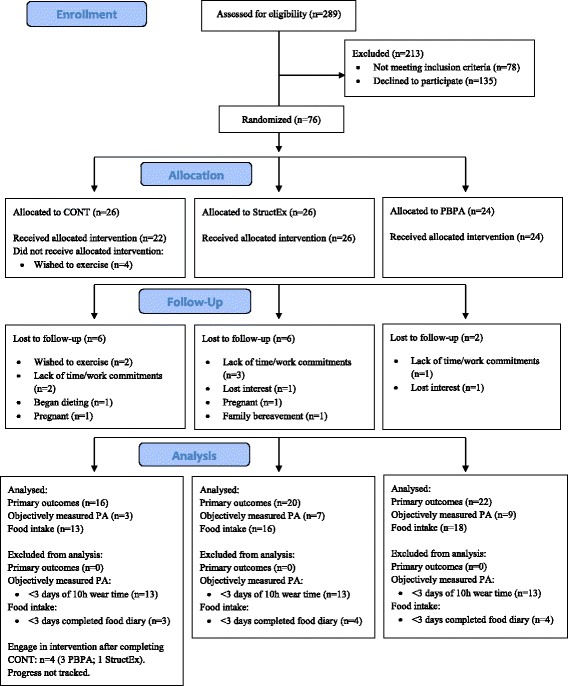
CONSORT Flow diagram of participation and analysis during the study

Of the 289 assessed for eligibility, 76 women participants (mean age 41 ± 2 years; mean BMI 29.2 ± 3.4 kg•m^− 2^) were recruited from the West Midlands, UK and admitted to the study (Fig. 1). Inclusion criteria were: age 21 to 50 years; a BMI of 25-35 kg•m^− 2^; and being physically inactive (<150mins of self-reported MVPA per week). Exclusion criteria were: dieting or intent to diet; any musculoskeletal, metabolic or cardiovascular disorders; medication that influenced lipid metabolism or appetite; blood pressure > 140/90 mmHg; smoking; and pregnancy or breast-feeding. Participants meeting inclusion/exclusion criteria were given details of study procedures and provided their informed consent.

### Intervention

Participants in the StructEx condition were instructed to undertake 5 × 30 min of moderate-intensity exercise per week to achieve the weekly recommendations of 150 min of moderate-intensity exercise [34]. Through discussion with a member of the research team, considering exercise preference, perceived ability and feasibility (based on time, access to facilities and cost), participant identified a structured routine for completing 5 × 30 min of exercise each week to which they felt they would be able to adhere. All identified one or two modes of exercise to form their routine. Participants were advised on how to attain exercise of a moderate-intensity (i.e., “you should be able to talk, but not sing the words of a song” [35]).

Those in the PBPA condition were provided with a table of examples of different activities, each with a points score allocated per ten-minutes of activity (see Additional file 1). Points values were derived from MET scores [36], and adjusted for the MET score of 1.5 for sedentary behaviour. (Points score per 10 min of activity = MET score for that activity - 1.5. For example, the MET score for mowing the lawn is 5.5 METs; so the points score for mowing the lawn = 5.5–1.5 = 4 points). Participants were instructed to accumulate 30 points per week, equating to 5 × 30 min of brisk walking. This enabled the PBPA and StructEx conditions to be matched for MET-assigned PA-related energy expenditure. It was made explicitly clear to participants in both groups that activities should be of ≥10 min duration to contribute to their weekly points total, and these activities had to be additional to regular PA behaviour. In addition to the table of activities provided, participants in PBPA were asked what other forms of activity they may be interested in undertaking and points scores were provided for these activities. It was made clear to participants that they were not restricted to the activities in the table and those discussed at this preliminary visit; if they performed any additional type of activity, they were instructed to contact a member of the research team to receive a points score for that activity. Activities for which points scores were requested included chopping wood (4.5 points), painting and wallpapering (1.5), moving furniture (4.5), shopping (0.8) and bowling (1.5).

To encourage adherence, participants in PBPA and StructEx were contacted by telephone and email twice weekly for the first 4 weeks, and once fortnightly from weeks 4 to 12. Participants were asked of their adherence to the condition in which they were in. If target points/min were being achieved, positive reinforcement was offered. If not, then they were encouraged to persevere, and reminded of the typical benefits of being more active for health and general well-being. No contact was made during weeks 13 to 24, other than to arrange study visits.

Those in the CONT condition were instructed to maintain their current lifestyle for the 24-week study period. They were informed that they would be offered the opportunity to complete the intervention of their choice after the study period. All participants, in all three conditions, were instructed not to consciously change their dietary habits.

### Procedures and protocols

#### Pre-testing

Participants arrived at the School of Sport, Exercise and Rehabilitation Sciences, University of Birmingham for an initial visit. Following informed consent, measurements of height, weight and blood pressure were recorded. Participants were then randomly allocated to one of the three study conditions by draw of opaque, sealed envelopes. Participants were then given verbal instructions on how to accurately complete the three-day food diary and wear the activity monitor. A separate one-day food diary was administered to be completed the day before the baseline test day. Participants would be asked to replicate this intake the day prior to all subsequent test days, with one-day food diaries completed on the day before each trial visit to allow for a check of adherence to the dietary control. One week prior to the test day at week 24, the participant was contacted by email or telephone and food diary and activity monitor were collected by or posted to the participant.

#### Test-day protocol

Participants returned to the laboratory a minimum of 5 days after visit 1 for baseline measures. Test-day visits were repeated at weeks 4, 12 and 24. At each visit, participants arrived between 07:00 and 09:00 after a minimum 10 h overnight fast and after abstaining from exercise or alcohol consumption during the previous 24 h. Upon the negative result of a urine pregnancy test and after voiding, anthropometric measures of height, weight and waist and hip circumference were obtained. The dual-energy x-ray absorptiometry (DXA) scan for analysis of body composition was then conducted. Participants were then provided with a small glass of water and a cereal bar to break their fast. At baseline and week 24, food diaries and activity monitors were collected from participants.

### Outcome measures

#### Primary outcomes

##### Body composition

Body composition was assessed using the DXA method (Hologic Discovery QDR). Calibration and quality control checks were conducted prior to data collection for each trial visit. Values of whole-body fat mass (WBFM) and lean mass (WBLM), as well as measures of central adiposity were obtained. Android fat and abdominal visceral adipose tissue area (VAT) were measured across the abdomen in a 5 cm wide region between the iliac crest and the 4th lumbar vertebrae [37]. All values were calculated using the Hologic software programme (version 13.4.2).

##### Anthropometric measures

Body weight was assessed by semi-nude, post-void weighing using digital scales (Ohaus Champ II). Height was measured using a stadiometer (seca 220) and waist (at the level of the umbilicus) and hip (maximum posterior extension of the buttocks, level in the transverse plane) circumferences were measured using a tape measure (seca 201). Measurements were conducted by the same women researcher to maintain reliability. Circumferences were measured three times and a mean value calculated.

#### Secondary outcomes

##### Physical activity

Self-report PA was recorded by participants using a hard-copy activity diary. PA points and minutes of exercise were recorded by those in PBPA and StructEx, respectively, with weekly totals calculated for monitoring. Participants were encouraged to add each entry into the diary as soon after completing the exercise or activity as possible, clearly stating the type of activity or exercise, the duration and the points score for the activity, where relevant. Entries were checked for clarity and calculations checked for accuracy by a member of the research team at each visit to the laboratory. Objectively-measured PA was assessed using the GT3X accelerometer (Actigraph, FL). The GT3X accelerometer records movements measured over pre-specified time periods (epochs). Movements are summed to represent activity counts and interpreted to estimate frequency, intensity and duration of PA. In this study, accelerometers were initialised to measure PA over 15 s epochs. Sedentary time, light intensity PA and MVPA engagement were determined using cut-points as per Troiano and colleagues [38]. Participants were instructed to wear the accelerometer on their right hip during all waking hours for three consecutive days. Data was analysed from participants who provided a full 3 days of valid data (valid day = ≥10 h wear-time).

##### Food intake

Food intake was recorded using a 3-day weighed food diary (two weekdays and one weekend day). Prior to the collection of data, participants were provided with weighing scales and instructed how to accurately complete the diary. They were also provided with an example of a highly-detailed recording of intake. Participants were asked to avoid recording on days when it was expected that eating behaviour would be atypical. The completed food diaries were inspected upon collection from the participants and any foods or weights that were lacking detail or clarity were queried to help obtain necessary additional information. Food diaries were analysed using Dietplan (version 6.0) to gain mean daily intakes of total energy (kcal), carbohydrate, protein and fat (grams).

### Statistical analysis

Analyses were conducted using SPSS (version 22.0). Baseline differences for all variables were assessed using one-way ANOVA. Where significant differences were observed, change-from-baseline values were used. For variables where magnitude of change over time is of likely of specific interest (body weight and fat mass), change-from-baseline data was also presented and analysed. Mixed-design ANOVA with repeated measures were used to assess differences between groups from baseline weeks 4, 12 and 24 (body composition, body weight and waist circumference) and baseline to 24 weeks (objective PA and food intake). For objective PA, percentage times spent in different intensities of PA were used in analyses to adjust for variability in accelerometer wear. Bonferroni post-hoc tests were used to interpret significant main and interaction effects.

For primary outcome measures, missing data analysis using the multiple imputations techniques was conducted for missing data points at weeks 4 and 12. This was the case for 6 measures in total (2 control participants missing data at 4 and 12 weeks and 2 StructEx participants missing data at 12 weeks).

## Results

Of the 76 participants admitted to the study, 58 completed the intervention period and attended the 24-week follow up. The data of all 58 were analysed for all primary outcome measures. Data of 19 participants who provided ≥3 days accelerometer data (≥10 h·day^− 1^) were analysed for objectively measured PA and data of 41 participants were analysed for food intake (Fig. 1).

### Body composition

**Fig. 2:**
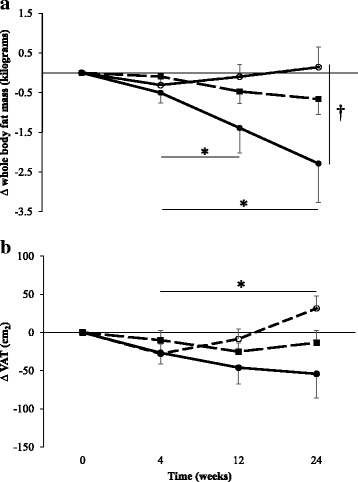
Changes in whole body fat mass (**a**), and visceral adipose tissue (**b**) from baseline at 4, 12 and 24 weeks in CONT (○), StructEx (■) and PBPA (●). For post-hoc comparisons, * denotes significant within-group difference, † denotes significant between-group difference

Absolute values for WBLM, WBFM, VAT and android fat are shown in Table 1 and change from baseline data is shown in Fig. 1. For WBFM, the condition x time interaction approached significance (*p* = 0.060, η^2^_p_ = 0.89). There was a significant time main effect (*p* = 0.036, η^2^_p_ = 0.69), although pairwise comparisons did not identify significant differences between specific time points. When analysing change-from-baseline data (Fig. 2a), there was a significant group x time interaction (*p* = 0.049, η^2^_p_ = 0.95), with a trend for a difference in change-from-baseline at 24 weeks for WBFM between CONT and PBPA (*p* = 0.075, d = 0.683, 95%CI: -5.04 – 0.17 kg). In PBPA, reduction in WBFM was significantly greater at 12 weeks and 24 weeks, compared with at 4 weeks (both *p* < 0.05). There was a significant group x time interaction for WBLM (*p* = 0.028, η^2^_p_ = 0.088). However, no significant post-hoc pairwise comparisons were present.

**Table 1:** Body composition and anthropometric measures for CONT, StructEx and PBPA at baseline, 4 weeks, 12 weeks and 24 weeks. Values are mean ± SD

	CONT				StructEx				PBPA			
	Base	4 weeks	12 weeks	24 weeks	Base	4 weeks	12 weeks	24 weeks	Base	4 weeks	12 weeks	24 weeks
WBFM (kg) * *L*	30.6 ± 8.1	30.3 ± 7.9	30.5 ± 8.5	30.8 ± 7.7	33.7 ± 6.3	33.6 ± 6.1	33.2 ± 6.2	33.0 ± 6.9	31.7 ± 6.7	31.2 ± 6.3	30.3 ± 6.1	29.4 ± 6.7
WBLM (kg) † *L*	43.4 ± 4.6	43.8 ± 4.8	43.7 ± 4.4	44.2 ± 4.2	46.1 ± 4.0	46.3 ± 4.4	46.5 ± 4.6	46.2 ± 5.3	44.4 ± 5.3	44.4 ± 4.8	44.3 ± 4.6	43.8 ± 5.0
VAT (cm^2^)	486 ± 238	458 ± 227	477 ± 245	517 ± 246	679 ± 208	669 ± 198	654 ± 205	665 ± 207	543 ± 195	517 ± 166	497 ± 172	489 ± 184
Android fat (kg) † *M*	2.30 ± 0.79	2.20 ± 0.78	2.28 ± 0.83	2.39 ± 0.85	2.88 ± 0.78	2.85 ± 0.73^d^	2.83 ± 0.79	2.80 ± 0.84	2.47 ± 0.76	2.36 ± 0.67	2.27 ± 0.64^a^	2.19 ± 0.74^a,e^
Body weight (kg) † *M*	76.9 ± 10.9	75.9 ± 11.3	76.2 ± 11.4	76.5 ± 10.9	81.0 ± 9.4	81.1 ± 9.6	80.6 ± 9.7	80.5 ± 11.2	77.5 ± 9.3	76.7 ± 8.5	75.8 ± 8.0	74.2 ± 8.7^a,b^
Waist circ. (cm)	79.5 ± 11.6	80.9 ± 8.5	81.4 ± 9.5	81.5 ± 8.9	88.8 ± 7.0^d^	88.8 ± 8.1	87.4 ± 7.6	88.0 ± 9.0	83.6 ± 7.6	83.0 ± 7.0	82.1 ± 6.4	80.8 ± 6.9
Waist:hip ratio	0.74 ± 0.09	0.78 ± 0.08	0.75 ± 0.07	0.76 ± 0.06	0.82 ± 0.04^d^	0.82 ± 0.05	0.82 ± 0.05	0.82 ± 0.05	0.78 ± 0.06	0.78 ± 0.06	0.78 ± 0.06	0.78 ± 0.06

*WBFM* whole body fat mass, *WBLM* whole body lean mass, *VAT* visceral adipose tissue

†, significant interaction; *, significant time main effect. *S*, small effect, 0.01; M, medium effect, 0.06; L, large effect, 0.14

^a^, significantly different from baseline; ^b^, significantly different from 4 weeks; ^c^, significantly different from 12 weeks; ^d^, significantly different from CONT group; ^e^, significantly different to StructEx

Effect sizes and significance are not shown for VAT, waist circumference and waist:hip ratio due to significant differences at baseline

Values for VAT differed significantly between groups at baseline. Therefore, change-from-baseline values are included and used for analysis (Fig. 2b). There was a significant group x time interaction for VAT change-from-baseline (*p* = 0.024, η^2^_p_ = 0.103). The difference in change-from-baseline between CONT and PBPA approached significance at 24 weeks (*p* = 0.053, d = 0.743, 95%CI: -0.91 – 172.3 g). There was a significant group x time interaction for android fat (*p* = 0.004, η^2^_p_ = 0.137. Table 1) with mean values significantly lower at 12 weeks and 24 weeks compared with baseline in PBPA (*p* = 0.005, d = 0.285, 95%CI: -0.34 – -0.04 kg and p = 0.005, d = 0.373, 95%CI: -0.50 – -0.06 kg, respectively) and values significantly lower in PBPA than StructEx at 24 weeks (*p* = 0.048 d = 0.771, 95%CI: -1.23 – -0.40 kg).

### Anthropometric measures

**Fig. 3:**
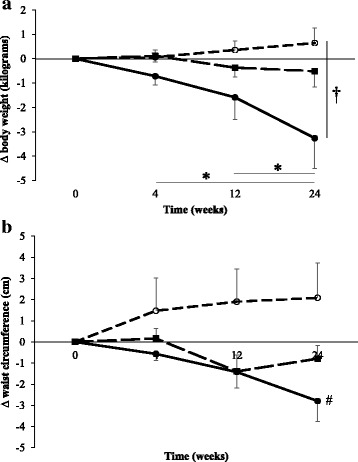
Changes in body mass (**a**) and waist circumference (**b**) from baseline at 4, 12 and 24 weeks in CONT (○), StructEx (■) and PBPA (●). * denotes significant within-group difference, † denotes significant between-group difference, # denotes significant group main effect: change significantly different between PBPA and CONT

Data for anthropometric measures are shown Table 1. There was a significant group x time interaction for body weight. Post-hoc analysis for within-group comparison showed that, in PBPA, body weight was significantly lower at 24 weeks compared with baseline (*p* = 0.004, d = 0.361, 95%CI: -5.73 – -0.78 kg) and 4 weeks (*p* = 0.008, d = 0.295, 95%CI: -4.59 – -0.50 kg). Comparisons between 24 weeks and 12 weeks approached significance (p = 0.053). There was a significant group x time interaction for change-from-baseline (*p* = 0.026, η^2^_p_ = 0.111. Figure 3a). At 24 weeks, change-from-baseline was significantly greater in PBPA compared with CONT (*p* = 0.020, d = 0.865, 95%CI: -7.38 – -0.49 kg). Within PBPA, body weight-change-from-baseline was significantly greater at 24 weeks compared with 4 weeks (p = 0.004, d = 0.587, 95%CI: -4.39 – -0.70 kg) and 12 weeks (*p* = 0.006, d = 0.325, 95%CI: -2.84 – -0.39 kg).

Waist circumference and waist-to-hip ratio differed significantly between CONT and StructEx at baseline. Therefore, change-from-baseline values were assessed. There was no significant group x time interaction effect (*p* = 0.832) for waist circumference but there was a significant main effect of group (*p* = 0.029, η^2^_p_ = 0.123). Change in waist circumference (Fig. 3b) was significantly greater in PBPA compared with CONT (*p* = 0.024, d = 0.677, 95%CI: 0.3–5.8). There were no significant interaction or main effects for waist-to-hip ratio (all *p* values> 0.05).

### Physical activity

Mean weekly minutes of self-reported exercise across the 24-week intervention period in StructEx was 172 ± 72 min. The mean weekly number of self-reported PA points reported was 39.1 ± 9.3, equating to 197 ± 54 min of brisk walking. When comparing self-reported minutes and self-reported minute equivalents, total activity reported did not differ between groups (*p* > 0.05).

Objectively measured PA is shown in Table 2. No significant main or interaction effects were observed from baseline to 24 weeks for sedentary time, light PA or MVPA (all p > 0.05). However, the time x group interaction effect for light PA approached significance (*p* = 0.066) and there was a trend for an interaction effect for sedentary time (*p* = 0.087). Interpretation of means indicated the PBPA condition increased light PA engagement and reduced sedentary time from baseline to 24 weeks, relative to StructEx and CONT conditions.

**Table 2:** Percentage of daily time spent in moderate and vigorous physical activity, light physical activity and sedentary in CONT, StructEx and PBPA at baseline and 24 weeks. Minutes of activity per day are included in parentheses, but not used for analyses. Values are mean ± SD

	CONT (*n* = 3)		StructEx (*n* = 7)		PBPA (*n* = 9)		Interaction *p* value	Effect size (η^2^_p_)
	Baseline	24 weeks	Baseline	24 weeks	Baseline	24 weeks		
MVPA (% time) *(minutes)*	6.12 ± 1.60 *(47.9 ± 10.9)*	4.58 ± 2.73 *(38.3 ± 23.0)*	4.94 ± 2.66 *(39.0 ± 21.8)*	7.78 ± 1.99 *(62.6 ± 17.8)*	5.09 ± 1.97 *(41.4 ± 17.5)*	5.95 ± 1.40 *(48.9 ± 12.7)*	0.100	0.250
Light PA (% time) *(minutes)*	25.3 ± 4.3 *(200.7 ± 43.4)*	21.8 ± 12.3 *(182.2 ± 103.7)*	19.9 ± 4.5 *(157.4 ± 40.4)*	18.8 ± 3.9 *(150.5 ± 33.7)*	23.4 ± 7.0 *(192.3 ± 58.8)*	27.3 ± 9.3 (222. *±73.9)*	0.066	0.288
Sedentary (% time) *(minutes)*	68.5 ± 2.73 *(539.9 ± 20.2)*	73.6 ± 12.8 *(614.0 ± 103.9)*	75.1 ± 6.42 *(589.3 ± 47.1)*	73.5 ± 5.0 *(588.3 ± 51.8)*	71.6 ± 7.92 *(587.5 ± 58.8)*	66.8 ± 9.1 *(546.8 ± 85.5)*	0.087	0.263

*MVPA* moderate-vigorous physical activity, *PA* physical activity

Data were only included where participants met minimum wear criteria (i.e., =3 days of valid data with ≥10 h wear time per day, *N* = 19)

### Food intake

Mean daily intakes of energy (kcal), carbohydrate, fat and protein at baseline and 24 weeks are shown in Table 3. There was a trend for a group x time interaction (*p* = 0.054, η^2^_p_ = 0.122), with reductions in energy intake observed in CONT (− 233 kcal) and PBPA (− 445 kcal), compared with little change in StructEx (− 52 kcal). There was a significant main effect for time (*p* = 0.001, η^2^_p_ = 0.219) with intake at 24 weeks lower than intake at baseline (d = 0.488, 95%CI: -384 – -106 kcal). There was a trend for group main effect (*p* = 0.061, η^2^_p_ = 0.177).

**Table 3:** Mean values for daily energy, carbohydrate, fat and protein intake for CONT, StructEx and PBPA at baseline and 24 weeks. Values are mean ± SD

	CONT (*n* = 13)		StructEx (*n* = 16)		PBPA (*n* = 18)		Interaction *p* value	Effect size (η^2^_p_)
	Baseline	24 weeks	Baseline	24 weeks	Baseline	24 weeks		
Energy (kcal) ^b^	2067 ± 339	1834 ± 346	2172 ± 553	2120 ± 665	2007 ± 441	1562 ± 525	0.054	0.122
CHO (g) ^b^	258 ± 56.8	226.2 ± 54.4	266 ± 75.0	264 ± 93.6	252 ± 91.5	185 ± 60.9	0.059	0.121
FAT (g) ^a^	77.6 ± 20.0	67.1 ± 19.1	79.3 ± 33.3	84.6 ± 33.9	79.0 ± 20.8	60.0 ± 25.6 ^c,d^	0.047	0.130
PRO (g) ^b^	79.2 ± 21.7	73.1 ± 19.6	84.1 ± 27.4	75.1 ± 17.6	73.3 ± 17.2	65.4 ± 25.1	0.929	0.003
% energy CHO	47.0 ± 7.5	46.2 ± 7.3	47.1 ± 10.9	47.9 ± 8.9	46.7 ± 7.5	44.8 ± 8.0	0.723	0.014
% energy FAT	33.5 ± 5.4	32.8 ± 5.7	32.5 ± 5.4	36.0 ± 6.5	35.3 ± 7.1	34.7 ± 8.6	0.255	0.060
% energy PRO	15.4 ± 3.7	15.9 ± 3.0	15.9 ± 3.92	15.0 ± 3.2	15.2 ± 4.3	16.8 ± 3.9	0.215	0.068

*CHO* carbohydrate, *FAT* fat, *PRO* protein

^a^significant interaction; ^b^ significant time main effect

^c^significantly different to baseline; ^d^, significantly different to StructEx

There was a significant group x time interaction for absolute daily fat intake (*p* = 0.047, η^2^_p_ = 0.130), with fat intake significantly lower at 24 weeks compared with baseline in PBPA (*p* = 0.006, d = 0.720, 95%CI: -5.8 – -32.2 g). Further, fat intake was significantly lower in PBPA compared with StructEx at 24 weeks (*p* = 0.035, d = 0.903, 95%CI: -1.3 – -47.9 g). There was a trend for a group x time interaction for absolute daily carbohydrate intake that approached significance (*p* = 0.059). No group x time interaction was observed for absolute daily protein intake. However, both carbohydrate and protein intake were significantly lower at 24 weeks relative to baseline across all groups (main effect for time, carbohydrate: *p* = 0.005, η^2^_p_ = 0.163; protein: *p* = 0.011, η^2^_p_ = 0.151). When assessing the contribution of energy from each macronutrient to total energy intake, there were no within-group or between group differences (all interactions and main effects, *p* > 0.1).

## Discussion

This study investigated the effect of a novel points-based PA programme on body composition and body weight in inactive middle-aged women, who were overweight or obese, over 24 weeks. No reductions in WBFM, VAT or android fat were achieved in StructEx. However, participants in the PBPA condition successfully reduced WBFM by 2.3 kg (− 6.6%). Perhaps more importantly, significant reductions in android fat of 10.1% and reductions in VAT of 5.8% were achieved. When accounting for the 8% and 4% increases in these two measures, respectively, in the CONT condition, this equates to substantial improvements after 24 weeks of points-based PA.

The reduction in android fat observed in the PBPA condition is considerably greater than previously seen with 12 weeks of moderate intensity exercise training in overweight adults [39], while the reduction in VAT was similar to that observed with 16 weeks of multimodal exercise [33]. Excess visceral adipose tissue and android fat contribute to metabolic disorders [40] and have both been identified as independent risk factors for metabolic syndrome (MS) [41] and cardiovascular disease (CVD) [42, 43]. As such, any reduction in VAT and android fat may contribute towards the prevention of these long-term health conditions.

The PBPA and StructEx conditions were successful at inducing weight-loss and avoiding weight-gain, respectively. Results therefore suggest a PBPA centred intervention may be more effective for encouraging changes in PA behaviour likely to contribute meaningfully towards weight-loss, relative to exercise interventions that promote the following of a structured routine with less choice and flexibility. This study provides some evidence to support public health messages, and advice from practitioners and health care professionals, that advocate accumulating PA through the adoption of a variety and range of activities.

The mean 24-week weight-loss among individuals in the PBPA condition was 3.3 kg, equating to a 4% reduction from baseline. The corresponding rate of weight-loss per week (0.136 kg∙week^− 1^) is comparable to that observed in more efficacious exercise interventions [6, 12]. Still, this remains a smaller reduction in weight than is typically achieved through dietary restriction, and diet plus exercise interventions of a similar duration [6]. The American College of Sports Medicine [44] indicate that improvements in chronic disease risk factors can be achieved with weight-loss of as little as 2–3% of total body weight. Thus, the 4% reduction in total body weight observed in the PBPA condition further support the utility of employing a PBPA intervention to encourage levels of PA engagement likely to contribute towards clinically meaningful benefit.

This point can be further illustrated when examining results pertaining to waist circumference. Waist circumference is an independent risk factor of CVD [45, 46], with an increase of 1 cm resulting in a 2% increase in relative risk of a CV event [47]. A medium effect for change in waist circumference resulted in a 2.8 cm, (3.4%), reduction in waist circumference in PBPA compared with a 2.1 cm, (3.8%), increase in CONT, while change was minimal in StructEx. A waist circumference of > 88 cm is associated with increased risk of CVD [48]. In PBPA, three of the five participants that were in this at risk category at baseline reduced their waist circumference to less than 88 cm at 24 weeks. In comparison, of the ten participants in StructEx that were at increased risk at baseline all ten remained so at 24 weeks.

Based on self-reported activity, both StructEx and PBPA appeared to be equally well adhered to. Mean values of minutes and points exceeded the target of 150 min and 30 points per week, respectively. However, whilst self-reported adherence was similar between intervention conditions, both self-report and accelerometer assessed PA data indicate the PBPA intervention may have encouraged higher levels of PA engagement, relative to the StructEx and control conditions. Upon examination of individual self-report values, only two participants in PBPA reported failing to average 30 or more points per week over the 24 week whereas 6 participants failed to achieve an average of 150 min per week in StructEx. Further, four participants withdrew from the study in StructEx, citing loss of interest and failure to adhere due to work commitments, whereas only two participants withdrew for these reasons in PBPA. Specifically, while not statistically significant, a mean difference equivalent to 25 min of PA per week was reported between groups, with greater activity in PBPA.

Accelerometer data revealed a trend towards an increase in light PA among participants in the PBPA condition from baseline to 24 weeks, relative to the StructEx and CONT condition for whom declines in light PA were observed. In addition, a trend was also observed for a reduction in sedentary time in PBPA, equating to a group mean of 42 min less of sedentary behaviour a day. With no observed change in MVPA, it is possible that participants in PBPA replaced sedentary time with time engaged in light activity. The health enhancing effects of light PA are becoming increasingly well documented [49–52], while sedentary behaviour has been identified as an independent risk factor for CVD [53–55]. For example, replacing sedentary behavior with light physical activity is reported to result in improvements in cardio-metabolic health (e.g., favourable changes in fasting plasma glucose, triglycerides and cholesterol [56]. This adds further support to the efficacy of utilising a PBPA approach to encourage PA behaviour change in order to improve broader health outcomes among middle-aged women, again supporting public health advice of adopting a variety of activities.

Nonetheless, differences in PA between the three groups do not completely reflect the changes in body composition and anthropometry. The decrease in sedentary time and an apparent increase in light activity alone cannot explain the reduction in body weight and fat mass seen in the PBPA group, and neither do differences in sedentary time, light activity and MVPA explain the differences between PBPA and StructEx in particular at 24 weeks. This study afforded measures of change in self-reported food intake in a free-living setting. While not statistically significant, mean self-reported energy intake reduced by 445 kcal from baseline to 24 weeks in PBPA. This was in comparison to a negligible reduction of 52 kcal in StructEx and a smaller reduction of 233 kcal in CONT. Further, mean self-reported energy intake at 24 weeks was 558 kcal lower in PBPA compared with StructEx: a difference that likely contributed to the observed significant group main effect. This difference appears driven by the significantly lower fat intake (171 kcal) and a trend for a lower carbohydrate intake (268 kcal) in PBPA. It has been claimed that an energy deficit of greater than 500 kcal•day^− 1^ is required for successful weight-loss [44, 57, 58], so it is likely that the observed reduction in energy intake contributed considerably to the weight loss achieved in PBPA, with changes in PA behaviour having a much smaller role.

It may have been expected that increased activity and subsequent energy deficit and reductions in fat mass would promote mechanisms to increase appetite and food intake [59]. However, conversely, food intake was reduced in PBPA. This may be an example of the “spill-over” effect whereby engagement in one type of health related behaviour has a positive influence on engagement in others [60]. It has been shown from cross-sectional data that active individuals are more likely to display other healthy behaviours [61] including reduced fat intake [62], while women who were overweight reduced total energy intake during 12 weeks of resistance exercise [63]. Interestingly, this was not seen with the StructEx intervention. It may be that encouraging participants to collect PA points throughout the day in numerous activity bouts could have enhanced or prolonged engagement in the health-related behaviour of PA, compared with StructEx. This may have raised consciousness for health-related behaviours, and hence formed a greater stimulus for a “spill-over” effect. Also, the greater early-stage positive changes in body weight and waist circumference observed in PBPA may have resulted in increased self-efficacy for the adoption of other health behaviours [60].

Interestingly, a recent study by Beer et al. [64] observed a greater energy intake, driven by a high prevalence of “unhealthy” food choices, after an acute bout of exercise when participants had no choice over the mode, intensity, duration and time of commencement of the exercise, compared with participants who had choice over these factors. This was despite no difference in subjective appetite ratings. The authors propose that choice resulted in a greater perception of autonomy with regards to their PA engagement, and that this may have also resulted in greater perceived self-regulation in another health-related context (i.e., diet), facilitating the selection of healthier food choices. It is possible that the same mechanism underpinned healthier food choices for participants in PBPA, compared with those in StructEx. As such, a points-based approach to PA, while having small, positive effects on activity and sedentarism, may infer meaningful and beneficial effects on other health behaviors, such as diet.

The approach of favouring measures obtained within a free-living setting was necessary to assess the effectiveness of the exercise and PA interventions of this study, going beyond simply testing efficacy within a controlled laboratory setting. This was deemed preferable for increased ecological validity and generalisabilty. As such, the data would suggest that a points-based approach to physical activity may be an effective strategy for free-living interventions in women who are overweight.

The present study is not without limitations. Secondary measures were obtained only at baseline and week 24. It would have been preferable to obtain and analyse data for secondary measures at weeks 4 and 12. However, whilst attempts were made to do so, protocol adherence for objectively assessed PA and food intake was low. Consequently, the final sample for secondary measures may have been underpowered to detect significant differences between groups in response to the intervention, particularly with regards to PA, so findings in relation to these measures should be interpreted with some caution. Further studies are therefore required to consolidate the findings reported herein for which promising trends were observed (e.g., increases in light PA, reduced sedentary time, reduced energy intake). Further, adherence to the PBPA and StructEx conditions, and dietary intake were measured using self-report methods. It is acknowledged that such approaches can result in misreporting and inaccuracy of data. However, preference was for free-living measures and the assessment of the effectiveness, rather than efficacy, of the interventions. Self-reported PA data was also supported with data on objectively measured PA at baseline and week 24. However, we acknowledge that compliance with accelerometer protocols was low (33%), and as such, firm conclusions regarding the role of interventions tested herein, cannot be drawn on the basis of this data. Still, results provide an initial indication of the relative efficacy of these interventions for promoting engagement in light, moderate and vigorous PA, and reducing sedentary time. Despite the long-held appreciation of the limitations of food diaries [65, 66], this approach is still commonly-used in weight-management research [8, 11, 28, 31, 32]. Recently proposed electronic dietary intake assessment approaches, utilizing technology to allow ecological momentary analysis [67] have shown promise [68], and warrant consideration for use in future research.

The novel points-based approach to PA in the present study demonstrates promise as a strategy for reducing fat mass, body weight, waist circumference, sedentarism and food intake. Nonetheless, it was not successful at maintaining lean mass during weight-loss. This may be because the PBPA condition did not elicit an increase in MVPA, which may be considered a limitation of adopting such an approach to a PA programme. In addition to the current approach, and while ensuring autonomy over exercise components, incorporation of some degree of high-intensity [69] or resistance exercise [70] may help maintain lean mass; it is tempting to speculate that the greater increase in MVPA in StructEx compared with PBPA might have contributed to the better maintenance of lean mass. Further, incorporation of dietary manipulation, specifically increasing protein intake, may also facilitate maintenance of lean mass and further promote fat loss, as was observed by Josse et al. [71]. Further research employing such strategies is warranted to investigate the long-term effectiveness of a points-based approach to for increasing the effectiveness of PA towards levels necessary for improved body composition and weight-loss.

## Conclusion

Findings suggest that a point-based approach to PA accumulation is an effective strategy for inducing modest but meaningful reductions in body weight and body fat in inactive women who are overweight and obese. This is likely a result of modest reductions in sedentary time, increases in light activity and of inducing a spill-over effect of altered eating behaviour and reduced energy intake. Consequently, a points-based system may prove a worthwhile consideration for healthcare professionals when administering exercise and PA strategies to tackle inactivity, sedentarism, and overweight and obesity.

## Additional file

Additional file 1: Table of activities provided to the participants, with allocated points per 10-min of activity. (PDF 219 kb)

## Abbreviations

ANOVA: Analysis of variance
BMI: Body mass index
CONT: Control group
CV: Cardiovascular
CVD: Cardiovascular disease
DXA: Dual-energy x-ray absorptiometry
MET: Metabolic equivalent
MS: Metabolic syndrome
MVPA: Moderate-to-vigorous physical activity
NRES: National research ethics service
PA: Physical activity
PBPA: Point-based physical activity condition
StructEx: Structured exercise condition
VAT: Visceral adipose tissue
WBFM: Whole-body fat mass
WBLM: Whole-body lean mass
η^2^_p_: Partial eta squared
