# Supplementary material for: Points-based physical activity: a novel approach to facilitate changes in body composition in inactive women with overweight and obesity
Source: BMC Public Health. 2018 Feb 17;18:261. doi: 10.1186/s12889-018-5125-2 (PMC5816513; doi:10.1186/s12889-018-5125-2)
Supplement: Supplementary file 1 — Table of activities provided to the participants, with allocated points per 10-min of activity. (PDF 219 kb) [file 12889_2018_5125_MOESM1_ESM.pdf]

Additional file 1. Table of activities provided to the participants, with allocated points per 10-minutes of activity.

	**Activity**	**Number of points collected after 10 minutes**
**More strenuous activity ↑**	Jogging (general)	5.5 points
	Swimming (light effort)	5.5 points
	Cycling (light , 10-12mph)	4.5 points
	Indoor exercise bike (100 Watts, light effort)	4 points
	Mowing lawn	4 points
	Dancing (aerobic)	3 points
	Badminton	3 points
	Washing or painting a fence (outdoors)	3 points
	Playing Golf (pulling clubs)	2.5 points
	Cycling (slow , 10mph)	2.5 points
	Water aerobics	2.5 points
	Gardening	2.5 points
	Playing with children (moderately active)	2.5 points
	Scrubbing floors, bathroom	2 points
	Walking 3-3.5 mph (brisk)	2 points
	Vacuuming	2 points
	Sweeping floors	2 points
	Dancing (ballroom, slow)	1.5 points
	Washing car or windows	1.5 points
	Automobile repair	1.5 points
	Tending to a child (standing)	1.3 point
	Tending to a child (sitting)	1 point
	Walking 2 mph (slow)	1 point
	Ironing	0.8 points
**↓ Lighter activity**	Cooking or preparing food (standing)	0.5 points
